# Evaluation of the Expression Levels of miR-21-5p and miR-429 Genes in Biopsy Samples from Patients with Oral Squamous Cell Carcinoma

**DOI:** 10.3390/diagnostics13071244

**Published:** 2023-03-25

**Authors:** Ata Garajei, Abdolamir Allameh, Mehdi Azadi, Azadeh Emami, Mostafa Atashbasteh, Melina Mostafavi, Bayazid Ghaderi, Francesco Inchingolo, Masoud Sadeghi, Santosh Kumar Tadakamadla, Hady Mohammadi, Jyothi Tadakamadla

**Affiliations:** 1 Department of Oral and Maxillofacial Surgery, School of Dentistry, Tehran University of Medical Sciences, Tehran 14399-55991, Iran; 2 Department of Head and Neck Surgical Oncology and Reconstructive Surgery, Cancer Institute, School of Medicine, Tehran University of Medical Sciences, Tehran 14197-33141, Iran; 3 Department of Clinical Biochemistry, Faculty of Medical Sciences, Tarbiat Modares University, Tehran 14117-13116, Iran; 4 Department of Anesthesiology, School of Medicine, Iran University of Medical Sciences, Tehran 14176-13151, Iran; 5 Tehran Medical Branch, Islamic Azad University, Tehran 14197-33171, Iran; 6 Cancer and Immunology Research Center, Research Institute for Health Development, Kurdistan University of Medical Sciences, Sanandaj 66177-13446, Iran; 7 Department of Interdisciplinary Medicine, School of Medicine, University of Bari “Aldo Moro”, 70124 Bari, Italy; 8 Medical Biology Research Center, Kermanshah University of Medical Sciences, Kermanshah 67144-15185, Iran; 9 Dentistry and Oral Health, Department of Rural Clinical Sciences, La Trobe Rural Health School, La Trobe University, Bendigo, VIC 3550, Australia; 10 Violet Vines Marshman Centre for Rural Health Research, La Trobe Rural Health School, La Trobe University, Bendigo, VIC 3550, Australia

**Keywords:** squamous cell carcinoma, oral cavity, microRNA, gene expression

## Abstract

Introduction: MicroRNAs (miRs) are a group of endogenous, non-coding, 18-24 nucleotide length single-strand RNAs that mediate gene expression at the post-transcriptional level through mRNA degradation or translational repression. They are involved in regulating diverse cellular biological processes such as cell cycle, differentiation, and apoptosis. The deregulation of miRs affects normal biological processes, leading to malignancies, including oral squamous cell carcinoma (OSCC). This study evaluates the expression level of miR-21-5p and miR-429 genes in biopsy samples from patients with OSCC and performs a comparison with controls. Materials and Methods: In this study, tissue samples were obtained from 40 individuals (20 OSCC patients and 20 healthy controls) to determine miR-21-5p and miR-429 expression using the ΔCT method and analyzed by the Mann–Whitney test. Results: The mean age of subjects in the control and patient groups was 47.15 and 53.8 years, respectively. According to the Mann–Whitney test, significant differences were observed in miR-21-5p (*p* < 0.0001) and miR-429 (*p* = 0.0191) expression levels between the two groups (*p* < 0.05). Conclusions: The expression of miR-21-5p, miR-429, and combined miRNAs in the OSCC group was significantly higher compared to the control group. As a result, changes in the expression of these biomarkers in cancerous tissues could potentially be considered as a marker for the early diagnosis of OSCC.

## 1. Introduction

Oral squamous cell carcinoma (OSCC) is the most common malignancy of the oral cavity; it accounts for about 95% of all cases of head and neck cancers. The etiology of OSCC is multifactorial, with the disease being more prevalent among males in the sixth and seventh decades of life with smoking and drinking habits [1,2]. Despite advances in surgery and radiotherapy to treat this malignancy, the 5-year survival rate has not increased significantly [3]. If detected in the early stages, the survival rate is between 60 to 90% [4,5]. Due to the recurrence rate of 15–33% in patients with OSCC, appropriate diagnostic methods to predict the risk of recurrence in patients are also very important [6]. However, biopsy and histopathological examinations are still the most sensitive and specific methods to diagnose this type of cancer [7,8]. In addition to individual genetic factors [9,10,11,12,13,14], tobacco and alcohol consumption, exposure to radiation, and other carcinogens can effect head and neck cancer occurrence and progression [15]; therefore, a combination of environmental and genetic factors contribute to OSCC [16].

MicroRNAs (miRs) are involved in various physiological processes and play an important role in cancer; in mammals, the activity of more than 60% of the genes encoding proteins is controlled by the involvement of these molecules [17]. MiRs, along with DNA methylation and histone acetylation, have been introduced as epigenetic mechanisms. Therefore, changes in miR expression can affect important biological processes such as proliferation, differentiation, and apoptosis [18]. More than half of the known miRs are located in fragile regions of chromosomes and are susceptible to chromosomal deletion, addition, and transfer in various diseases, including cancer [19]. MiRs are usually encoded by endogenous genes and can have suppressive effects on post-transcriptional regulation of their target genes by suppressing translation or the degradation of mRNA [20]. This can activate or block further downstream signaling pathways related to oral cancers. Differences in miR expression between normal tissues, potential cancers, and oral tumor specimens provide an opportunity for miR to be used as an independent prognostic marker [21,22].

Cancer is the result of cells deviating from the correct pathways of regulation, proliferation, and differentiation. Self-efficacy in growth signals, insensitivity to growth-inhibitory signals, avoidance of programmed cell death, unlimited proliferative potential, maintenance of angiogenesis and tissue invasion, and metastasis leads to the malignancy of cancer [23]. Increased expression of miR in cancer cells may be due to the overexpression of a transcription factor or the depletion of CG islets in the promoter regions of the gene [24]. Recently, studies in Iran have examined the expression of several miRs in OSCC patients and their results have shown their expression to be associated with OSCC pathogenesis [25,26]. A review [27] reported that a wide variety of dysregulated miRs contribute to progression, pathogenesis, and specific outcomes of OSCC. Additionally, specific changes in the miR expression profile can transform a normal cell into a cancer cell [28,29]. Basing on the bioinformatics studies [30,31,32], two miRs, miR-21-5p and miR-429, were selected for evaluation of their expression in tissue samples of patients with OSCC in comparison to healthy individuals in Iran, and we thereby intend to explore the role of miR-21-5p and miR-429 as diagnostic biomarkers of OSCC.

## 2. Materials and Methods

### 2.1. Study Procedure

In this case-control study, 20 patients with OSCC and 20 healthy individuals were selected between August 2017 and August 2018 to assess the expression of miRs. Participants were selected from patients who were referred to the Shariati and Sina Hospitals and the Imam Khomeini Cancer Institute (Tehran, Iran). Patients who either had a documented diagnosis of OSCC based on physical examination and histopathologic evaluation were included in the case group. Participants in the control group were selected from patients who were referred to the dental department for extraction of impacted wisdom teeth. Participants were fully informed about the objectives of the study, voluntary participation in the study, and data confidentiality. Biopsies were taken from the affected areas of the mouth, tongue, or gums among participants with OSCC. Dental follicle samples associated with the impacted molars were collected from participants belonging to the control group. Tissue samples of the patients were collected from different areas of tumor tissues of the oral mucosa, including the tongue, gums, and other areas during resection surgery.

### 2.2. Inclusion Criteria

For patients with OSCC, the main inclusion criterion was the documented histopathologic diagnosis of OSCC by oral biopsy. For healthy individuals, the main inclusion criteria were impacted wisdom teeth and a lack of a history of malignancy and pre-malignant lesions such as leukoplakia, erythroplakia, and erythroleukoplakia. The minimum age of the participants was 18 years.

### 2.3. Exclusion Criteria

Patients with a history of receiving treatments such as chemotherapy and radiotherapy or any adjuvant treatment were excluded from this study. In addition, for all participants, any history of systemic, acute, or chronic inflammatory diseases was an exclusion criterion.

### 2.4. Total RNA and miRNA Extraction

Fresh tissue samples were cut into 0.5 cm sections and immediately placed in tubes containing 0.5 mL RNAlater^®^ buffer (Ambion, Austin, TX, USA) and then stored in a refrigerator at 4 °C for one day to allow the buffer to penetrate the tissue. RNAlater was extracted using the miRNeasy mini kit (Qiagen, Hilden, Germany). Total RNA extraction, including miR, was performed according to the manufacturer’s instructions. The quality and quantity of the extracted RNA were evaluated using a nanodrop spectrophotometer (2000c, Thermo Fisher Scientific, Wilmington, DE, USA) at 260 mm and 270 mm wavelengths. In general, the concentration of extracted RNAs was between 200 and 1000 ng/μL. The 280 nm wavelength is related to protein and the 260 nm wavelength is related to nucleic acid. The ratio of the 260/280 wavelengths indicates the ratio of nucleic acid to protein and this ratio was approximately 2.13. The samples were then frozen at −80 °C until evaluation.

### 2.5. cDNA Synthesis and qRT-PCR

The single-stranded cDNA was synthesized after RNA extraction. Then, the expression of miR-21-5p and miR-429 was assessed based on the guidelines by the miRCURY LNA™ Universal RT miR PCR manufacturer (Exiqon, Vedbaek, Denmark). cDNA synthesis and gene expression have been reported in our previous study [25]. The real-time quantitative polymerase chain reaction (qRT-PCR) primers were as follows: miR-21-5p forward: 5′-TAGCTTATCAGACTGATG-3′ and reverse: 5′-CAGTGCGTGTCGTGGAGT-3′; and miR-429 forward: 5′-AGGTCT CTGAGGGTCAAGCA-3′ and reverse: 5′-CTGGTTGAAAAGCATGAGCA-3′. Expression of miRs was shown based on the threshold cycle (CT) and the relative change or the fold change in expression was calculated using 2^−ΔCT^ = [(CT gene of interest − CT internal control) after normalization with the reference gene.

### 2.6. Statistical Analysis

The 2^−ΔΔCT^ equation was used to assess the desired miR expression in tumor tissue in patients in relation to normal tissue in controls. The distribution of age and sex was expressed by the mean and standard deviation for age and number of cases (percentage) for sex, respectively but the expression of genes was presented as median (min, max). The distribution of data normality was verified with the Kolmogorov–Smirnov test. The *t*-test and chi square/Fisher exact test were used to compare the age and sex distribution between the two groups, respectively. Mann–Whitney test was used to compare gene expression in patients and controls. In addition, Pearson’s correlation tests were conducted to explore the association between miRs. The difference in medians was estimated using the methodology of Hodges–Lehmann. Receiver operating characteristic (ROC) for the diagnostic values and area under the ROC curve (AUC) as a critical diagnostic index were evaluated to explore the potential clinical usefulness of tissue miRs expression level. A *p*-value less than 0.05 was statistically significant. All tests were two-sided or two-tailed. For sample size calculation, we relied on the estimates from a past study [33] to estimate a sample size for a study involving two independent groups with ‘mean’ as primary endpoint. A power of 90% and significance level of 5% were considered for sample size calculation. A sample size of 40 (20 cases for each group) was calculated to be adequate. All statistical computations were performed with SPSS^®^ 22.0 (IBM Corporation, Armonk, NY, USA) for Windows^®^.

### 2.7. Ethical Considerations

In this study, all participants signed a written informed consent form. The ethical approval was obtained from Tehran University of Medical Sciences, Tehran, Iran. In addition, the details received from the patients were kept anonymous.

## 3. Results

The demographic information of the subjects is given in Table 1. As can be seen, the mean age of the subjects in the case and control groups was 53.00 and 47.15, respectively. In this study, there were 11 men and 9 women in both groups. There was no significant difference between the two groups in terms of mean age and sex distribution (*p* > 0.05).

### 3.1. Evaluation of the Expression (ΔCT) of miR-21-5p and miR-429 in the OSCC and Control Groups

**Figure 1:**
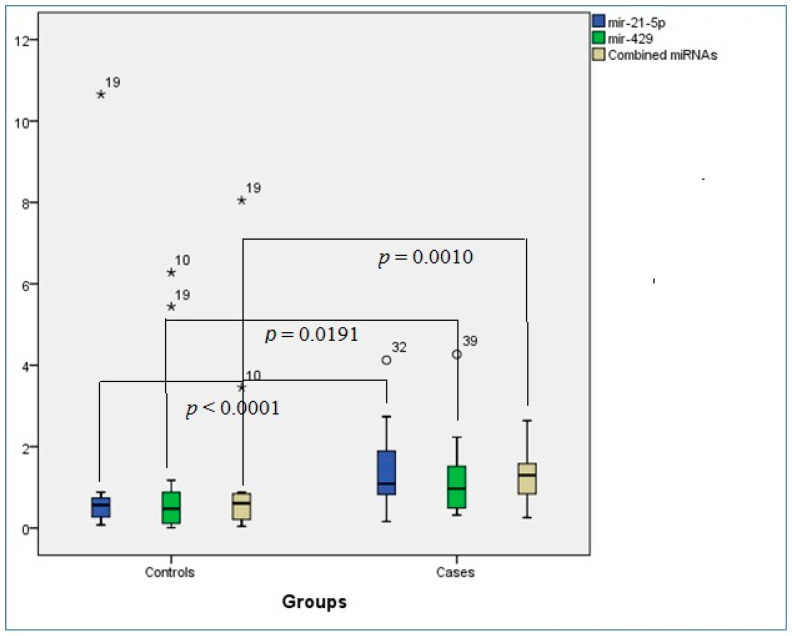
Distribution chart for the median expression of miRNAs among oral squamous cell carcinoma and control groups. Asterisk indicates outliers among controls and circle demonstrates outliers in the case group.

As can be seen in Table 2, the median ΔCT in the miR-21-5p expression was 0.57 in the control group and 1.09 in the case group; a significant difference (*p* < 0.0001) in the expression of miR-21-5p was observed between case and control groups. The median ΔCT for miR-429 expression in the control group (0.47) was significantly lower (*p* = 0.0191) than in the case group (0.97). In addition, the median ΔCT in the combined miRs expression was 1.29 in the case group and 0.61 in the control group; a significant difference (*p* = 0.0010) in the expression of miR-21-5p was observed between case and control groups. The boxplot chart for the median expression of miRNAs among the OSCC and control groups is shown in Figure 1.

### 3.2. Correlational Associations between the Expression of miR-21-5p and miR-429 Together

Table 3 shows correlations between the expression of miR-21-5p, miR-429, and combined miRs. The results showed a positive correlation between miR-21-5p and miR-429 (*p* < 0.01), miR-21-5p and combined miRs (*p* < 0.01), and miR-429 and combined miRs (*p* < 0.01). However, the strength of the relationship between combined miRs with each of miR-21-5p (r = 0.902) and miR-429 (r = 0.834) separately was more than the strength of the relationship between miR-21-5p and miR-429 (r = 0.514).

### 3.3. Sensitivity and Specificity

**Figure 2:**
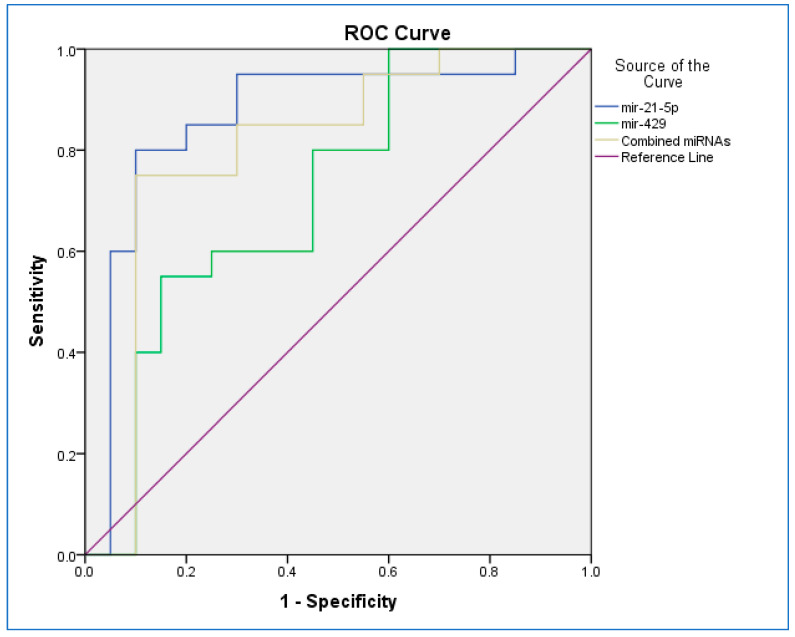
The receiver operating characteristic (ROC) curve for miR-21-5p and miR-429 and combined miRNAs.

The ROC curve was used to evaluate the sensitivity and specificity of two miRs (Figure 2). The ROC curves of the expression of miR-21-5p, miR-429, and combined miRs (miR-21-5p/miR-429) revealed their probability as valuable biomarkers with AUCs of 0.867, 0.715, and 0.805, respectively. The ROC curve revealed that, based on AUC, the expression of miR-21-5p had the highest sensitivity and specificity, and the least sensitivity and specificity were observed for the expression of miR-429. The cutoffs for the expression of miR-21-5p, miR-429, and combined miRs were 0.811, 0.854, and 0.875, respectively. Therefore, the diagnostic value of the expression of miR-21-5p was better than the expression of the combination of miRs or miR-429 alone.

## 4. Discussion

Oral cancer diagnosis requires methods with proven sensitivity and specificity that are operator-independent and can be repeated in cases of high-risk patients or in those treated cases who require a long follow-up period with a high likelihood for recurrence [34]. Despite the numerous methods of diagnosis, biopsy remains the gold standard in the diagnosis of OSCC [34]. The present study evaluating the tumor tissues from Iranian patients with OSCC revealed that miR-21-5p and miR-429 expressions were significantly higher in OSCC patients compared to controls, and that the combined miRNAs expression was higher in OSCC patients compared to controls. The results of the present study add to the current literature because these initial findings about changes in miR expression are important in better understanding the target molecules and their downstream pathways in the OSCC.

Nowadays, with social and industrial changes, the pattern of diseases has changed. Cancer is one of the leading causes of disorders, mortality, and disability worldwide. If lesions that are at risk of developing into cancer are identified and treated early, the patient may be relieved of the complications of cancer in addition to having a normal life expectancy and treatment [35]. In addition to genetic and environmental factors, epigenetic factors are also involved in the etiology of cancer. According to recent studies, interesting pathways for regulating gene expression have been identified that are mediated by small mRNA. These pathways include gene silencing, DNA methylation, gene transcription, and RNA interfering. MiRs are a large subset of non-coding RNAs of 18–25 nucleotides [36]. MiRs can act as oncogenes or suppress tumors and are involved in growth, programmed death, differentiation, and cell proliferation [18]. Cancer could result from a disorder in the regulation, proliferation, and differentiation pathways of cells [23].

Many miRs are abnormally expressed in cancer specimens. Many changes occur in cancer cells that can affect miR expression in a direct or indirect way. Abnormalities in miR genes or the proteins involved in their production, disorders in miR epigenetic regulation, and gene mutations are examples of these changes. However, the presence of miR in fragile areas is itself an important factor in altering miR expression in tumor cells. In addition, under the influence of mutations, the binding properties of miR and mRNAs are altered, and these altered miR/mRNA interactions lead to impaired translation processes [24]. Research on Iranian individuals examining miR-486-3p, miR-561-5p, miR-548-3p, and miR-509-5p [25] reported that miR-486-3p and miR-561-5p expressions were significantly lower, but that miR-548-3p expression was higher in 17 OSCC patients in comparison with 17 controls. In a study including 92 healthy individuals and 74 cases of OSCC [37], the authors found that there was a significant association between miRs expression and OSCC risk.

A study [38] showed that miR-21 was implicated in susceptibility to OSCC in 81 patients with OSCC. Another study [39] on 40 tissue samples of patients with OSCC and 40 samples of normal tissue showed that the expression of miR-21, miR-155, miR-191, and miR-221 increased significantly in people with OSCC. Liu et al. [40] also found that miR-31 is involved in patients with OSCC and there is a significant association between miR-21 and susceptibility to OSCC [41]. Aghiorghiesei et al. [42] revealed upregulation of miR-21-5p in OSCC cases under effect of tumor stage, and they [42] also showed that the identification of miR-21-5p alteration could be a useful target for the clinical application in OSCC. A meta-analysis [43] recently recommended that miR-21 can be a prognostic indicator of oral carcinoma. The present study showed a high expression of miR-21-5p in the OSCC compared to the controls. Therefore, miR-21-5p expression can be a suitable biomarker for OSCC diagnosis and progression.

MiR-429—a member of the miR-200 family—also has the potential to be used as a biomarker and might have relevance for the diagnosis, treatment, and pathogenesis of certain cancers in the future [44]. Another study identified the tumor suppressor role of miR-429 in OSCC [45]. The present study showed a high expression of miR-429 in the OSCC compared to the controls, demonstrating that miR-429 is another potential biomarker for OSCC.

The present study reported a stronger expression of combined miRs (miR-21-5p/miR-429) in the OSCC cases compared to the controls, particularly miR-21-5p and miR-429. Therefore, miR combination could be a more promising tool for cancer diagnosis in some situations and depending on the type of miRs. For example, a study [46] reporting hsa-miR-221 and hsa-miR-29c established a combination of two miRs as a promising tool for hepatocellular carcinoma diagnosis. Another study [47] recommended that the diagnostic power of the three miRs combined (hsa-mir-383, hsa-mir-615, and hsa-mir-877) was superior to that of a single miR in head and neck squamous cell carcinoma.

The present research had two important limitations. First, this study was of retrospective design and findings need to be confirmed through prospective clinical research. Second, we could examine only two miRs because of limited resources. In contrast, the strength of this study was that we matched the cases and controls based on age and sex.

## 5. Conclusions

The expression of miR-21-5p and miR-429 in the OSCC group was significantly higher than the control group. The diagnostic value of miR-21-5p expression was better than the expression of miR-429 or a combination of miRs for the detection of OSCC. However, a stronger expression of combined miRs in the OSCC cases was observed compared to the controls than miR-21-5p and miR-429 alone. These findings demonstrate the potential of miRs in the diagnosis of cancers and provide new data for the development of targeted molecular therapies for oral cancers. It is also suggested that the presence of these markers in the saliva and serum is investigated in future studies with bigger sample sizes, to explore their usability as a tool forFor OSCC screening, determining prognosis and measuring response to treatment, and also for early diagnosis of disease recurrence.

## Figures and Tables

**Table 1:** Demographic characteristics of the subjects.

Variable	Case (n = 20)	Control (n = 20)
Age, (years ± SD)	53.00 ± 11.80	47.15 ± 13.34
Sex, n (%)		
Male	11 (55)	11 (55)
Female	9 (45)	9 (45)

**Table 2:** Distribution [median (min, max)] of the expression of miRNAs among OSCC and control groups.

Variable	OSCC Group (n = 20)	Control Group (n = 20)	Actual Difference between Medians	Hodges–Lehmann Estimate	*p*-Value *
miR-21-5p	1.09 (0.16, 4.12)	0.57 (0.07, 10.65)	0.52	0.59	<0.0001
miR-429	0.97 (0.32, 4.27)	0.47 (0.01, 6.28)	0.50	0.42	0.0191
Combined miRs	1.29 (0.26, 2.64)	0.61 (0.04, 8.04)	0.68	0.66	0.0010

* based on Mann–Whitney test.

**Table 3:** Correlation between the expressions of miRNAs.

	miR-21-5p	miR-429	Combined miRs
miR-21-5p	-	0.514 **	0.902 **
miR-429	-	-	0.834 **
Combined miRs	-	-	-

Notes: ** = *p* < 0.01.

## Data Availability

The datasets used and/or analyzed during the current study are available from the corresponding author on reasonable request.

## Abbreviations

OSCC: oral squamous cell carcinoma; miR: microRNA; qRT-PCR: quantitative real-time polymerase chain reaction, ROC: receiver operating characteristic; AUC: area under the curve.
